# The Proto-Oncogene LRF Is under Post-Transcriptional Control of MiR-20a: Implications for Senescence

**DOI:** 10.1371/journal.pone.0002542

**Published:** 2008-07-02

**Authors:** Laura Poliseno, Letizia Pitto, Marcella Simili, Laura Mariani, Luisa Riccardi, Alessia Ciucci, Milena Rizzo, Monica Evangelista, Alberto Mercatanti, Pier Paolo Pandolfi, Giuseppe Rainaldi

**Affiliations:** 1 Laboratory of Gene and Molecular Therapy, Institute of Clinical Physiology, CNR, Pisa, Italy; 2 Cancer Genetics Program, Beth Israel Deaconess Cancer Center, Department of Pathology, Beth Israel Deaconess Medical Center, Harvard Medical School, Boston, Massachusetts, United States of America; 3 Cancer Genetics Program, Beth Israel Deaconess Cancer Center, Department of Medicine, Beth Israel Deaconess Medical Center, Harvard Medical School, Boston, Massachusetts, United States of America; 4 Istituto Toscano Tumori, Firenze, Italy; National Cancer Institute at Frederick, United States of America

## Abstract

MicroRNAs (miRNAs) are short 20–22 nucleotide RNA molecules that act as negative regulators of gene expression via translational repression: they have been shown to play a role in development, proliferation, stress response, and apoptosis. The transcriptional regulator LRF (Leukemia/lymphoma Related Factor) has been shown to prevent p19ARF transcription and consequently to inhibit senescence in mouse embryonic fibroblasts (MEF). Here we report, for the first time, that LRF is post-transcriptionally regulated by miR-20a. Using a gene reporter assay, direct interaction of miR-20a with the LRF 3′UTR is demonstrated. To validate the interaction miR-20a/3′UTR LRF miR-20a was over-expressed, either by transient transfection or retroviral infection, in wild type mouse embryo fibroblasts and in LRF-null MEF derived from LRF knock-out mice. We observed LRF decrease, p19ARF increase, inhibition of cell proliferation and induction of senescence. The comparison of miR-20a activity in wt and LRF-null MEF indicates that LRF is the main mediator of the miR-20a-induced senescence and that other targets are cooperating. As LRF down-regulation/p19ARF induction is always accompanied by E2F1 down-regulation and increase of p16, we propose that all these events act in synergy to accomplish miR-20a-induced senescence in MEF. Senescence has been recently revaluated as a tumor suppressor mechanism, alternative to apoptosis; from this point of view the discovery of new physiological “senescence inducer” appears to be promising as this molecule could be used as anticancer drug.

## Introduction

Pok proteins are a group of bifunctional proteins with potential oncogenic properties. They consist of an N terminal Poz domain and a C terminal kruppel-type (C2H2) zinc finger domain. The C-terminal zinc finger mediates specific DNA recognition and binding, while the amino-terminal POZ domain recruits histone deacetylase repressing transcription [1]. The oncogenic members of the Pok family include PLZF (Promyelocytic Leukemia Zinc Finger), whose t(11∶17) translocation is at the basis of acute promyelocytic leukaemia, [2], [3] and BCL-6 (B cell Lymphoma 6), which is deregulated in many types of lymphoma [4]. Another member of the family is LRF (Leukemia/lymphoma Related Factor) encoded by the *zbtb7a* gene and localized on chromosome 19p13.3 which is a hot spot for chromosomal translocations in human tumors. This protein, already known to have pleiotropic functions during embryogenesis [5], [6], [7], has recently been shown to act as an oncogene. When LRF is over-expressed in B and T lymphoid lineages (*lckEµ*-LRF), mice develop aggressive lymphomas that cause death between 9 and 40 weeks of age [8]. Like the other members of the family, LRF is a transcriptional repressor and p19ARF has recently been found to be a specific target in MEF [9]. p19ARF oncosuppressor is known as a key mediator of cell senescence. It mainly acts by inhibiting MDM2 and in turn up-regulating p53 levels [10]. LRF over-expression keeps p19ARF level down,preventing the onset of the senescence program and allowing single oncogenic proteins such as Ras to transform MEF [9].

Although it has been shown that LRF is over-expressed in some human cancers [8], little is known about the mechanisms causing its up-regulation.

MicroRNAs (miRNAs) have recently come into focus as a novel class of post-transcriptional regulatory elements. They are abundant endogenous ∼22-nucleotides (nt) RNAs that repress mRNA translation by base pairing to 3′UTR sequences [11]. The miRNA-target recognition is mainly due to the perfect complementarity between a short 7–8 nt stretch at 5′ end of the miRNA molecule (miRNA seed) and the corresponding stretch on the 3′UTR of the target gene (seed match). This results in a reduced translation and in turn a decreased level of the protein [12].

Some of the most studied microRNAs involved in human cancer are those belonging to miR-17-92 cluster. This cluster is located in a region amplified in lymphoma [13], [14] and lung cancers [15] while in other types of cancer, including nasopharyngeal carcinoma [16], [17], hepatocellular carcinoma [18] and breast cancer [19], the genomic location of the miR-17-92 cluster undergoes loss of heterozygosity suggesting that it may behave both as oncogene and oncosuppressor.

Enforced expression of the miR-17-92 cluster along with c-Myc expression accelerates tumor development in a mouse B-cell lymphoma model [13]. The underlying mechanism of action appears to be the E2F1 down-regulation by two members of the cluster, miR-17 and miR-20a. This c-Myc/miR-17-92 circuit helps to maintain E2F1 protein level below a pro-apoptotic threshold and cell proliferation prevails [20], [21], [22], [23].

On the other hand, in breast cancer cells the most relevant target of miR-17 is not E2F1, but AIB1. The nuclear receptor coactivator, amplified in breast cancer 1 (AIB1), acts as an oncogene. It actually enhances the transcriptional activity of many transcription factors, among which the estrogen receptor. Indeed, when miR-17 is over-expressed in breast cancer cells, a big decrease in ER-mediated signaling and in turn proliferation is observed and cells loose their ability to form colonies in soft agar [24].

Here, we report a novel activity exerted by miR-20a in mouse embryonic fibroblasts (MEF). This microRNA is able to control LRF protein at the post-transcriptional level and, when over-expressed, it induces premature senescence. This effect appears to be due to p19ARF up-regulation accompanied by up-regulation of p16 and down-regulation of E2F1.

## Results

We utilized early passages MEF, known to express LRF [9], to investigate whether this anti-senescence gene is regulated by miRNAs.

### miR-20a regulates LRF protein at the post-transcriptional level

**Figure 1:**
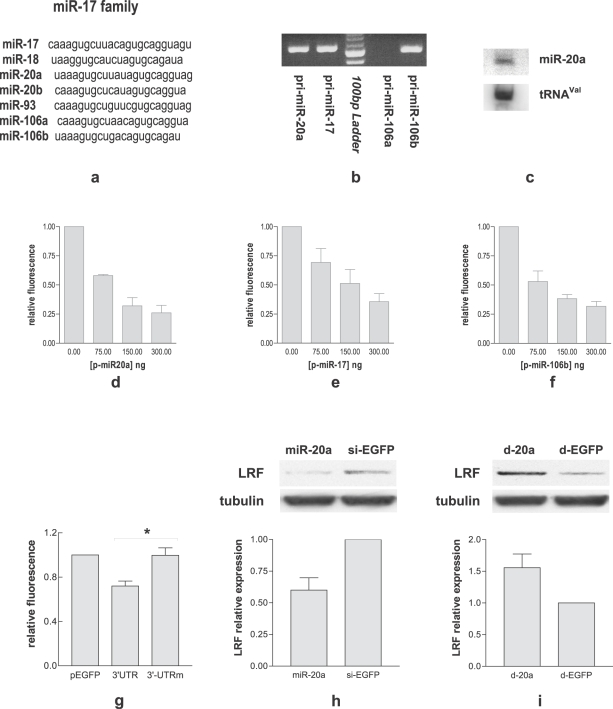
miR-20a regulates LRF expression in MEF. a: Sequences of miRNAs belonging to miR-17 family; b: RT-PCR analysis of pri-miRNA expression in MEF. Total RNA was extracted and amplified by RT-PCR using appropriate primers. The PCR products of ∼500 bp length are pri-miRNAs of the miR-17 family; c: Northern blot analysis of mature miR-20a expression in MEF. 20 µg of total RNA was analyzed with miR-20a probe or valine tRNA control probe; d, e, f: Interaction between 3′UTR of *mmu*-*zbtb7a* mRNA and miR-17 family. HEK293T cells were co-transfected with p-*zbtb7a* 3′UTR and increasing concentrations of p-miR-20a, p-miR-17 p-miR-106b or p-miR-26a control plasmid. 24 hours after transfection, cells were collected and the EGFP fluorescence intensity of each sample was determined with a FACscan analyzer. The relative expression of p-*zbtb7a* 3′UTR was obtained by the ratio of the mean fluorescence value of HEK293T cells transfected with p-miR-20a, p-miR-17 or p-miR-106b and the mean fluorescence value of HEK293T cells transfected with p-miR-26a control plasmid. Each bar represents the mean±SE of three independent experiments; g,: Transfection of p-miR-20a with either p-*zbtb7a* 3′UTR or p-*zbtb7a* 3′UTR_m_ in HEK293T. Cells were collected 24 hours after transfection and the EGFP fluorescence intensity of each sample was determined with a FACscan analyzer. The relative expression was obtained by the ratio of the mean fluorescence values of HEK293T cells cotransfected with p-miR-20a and either p-*zbtb7a* 3′UTR or p-*zbtb7a* 3′UTR_m_ normalized to that of HEK293T cells transfected with pEGFPC1. Each bar represents the mean±SE of three independent experiments. h i: Effects of over-expression/depletion of miR-20a on LRF expression.

*In silico* analysis with TargetScanS (http://genes.mit.edu/targetscan/) indicated various miRNA families potentially targeting the mouse *zbtb7a* 3′UTR. We focused our attention on the miR-17 family whose members are reported in Figure 1a [25]. Before testing if mouse *zbtb7a* 3′UTR interacts with miR-17 family members, the expression of representative members of the family was ascertained by RT-PCR. As reported in Figure 1b, MEF express the precursor of miR-20a, miR-17 and miR-106b, while they do not express the precursor of miR-106a. We then tested whether mouse *zbtb7a* 3′UTR interacts with miR-20a, miR-17 and miR-106b, using an EGFP reporter assay. miRNA-expressing plasmids (p-miRs) were investigated for their ability to inhibit fluorescence and it was found that p-miR-20a (Figure 1d), p-miR-17 (Figure 1e) and p-miR-106b (Figure 1f) all inhibit in a dose dependent manner.

Since we decided to focus on miR-20a throughout the experiments, the presence of the mature form in MEF cells was first ascertained by Northern blot (Figure 1c). To demonstrate the direct interaction, we again used the EGFP reporter assay. p-miR-20a was tested against p-*zbtb7a* 3′UTR wild type or mutated at the two binding sites specific for miR-17 family. p-miR-20a/p-*zbtb7a* 3′UTR interaction reduced the fluorescence, while p-miR-20a/p-*zbtb7a* 3′UTR_m_ interaction rescues the inhibition (Figure 1g) indicating that miR-20a binds directly *zbtb7a* 3′UTR.

Gain and loss of functions experiments were then performed. We observed that miR-20a over-expression, by transfection of 80 nM mature miR-20a, reduces LRF protein by 40% (Figure 1h). Conversely, inhibition of endogenous miR-20a by transfection with 80 nM antisense 2′-O-methyl-oligoribonucleotide (decoy, d-20a) increases LRF protein level by 55% (Figure 1i).

### miR-20a over-expression induces p19ARF

**Figure 2:**
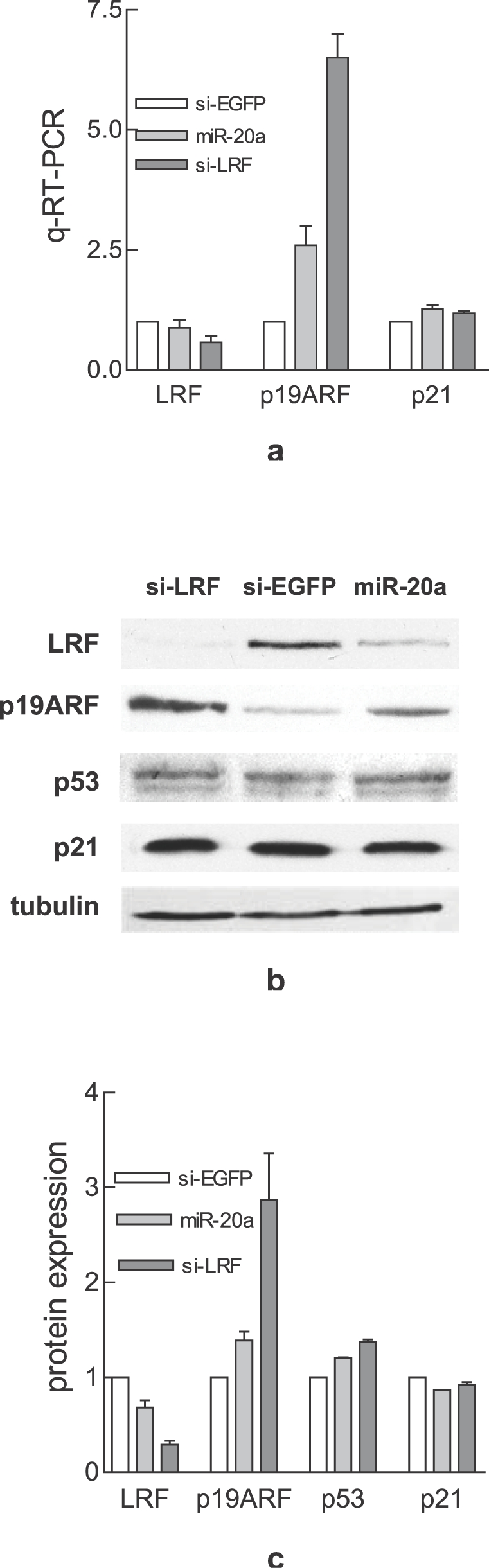
Effects of miR-20a on LRF-p19ARF pathway. a: q-RT-PCR of LRF, p19ARF and p21. Total RNA was extracted from MEF transfected with miR-20a, si-LRF or si-EGFP. mRNA level, detected by Real-Time PCR, was normalized to that of GAPDH. The values are the mean of two independent experiments. Western blot (b) and quantification (c) of LRF, p19ARF, p53 and p21. The band intensity was normalized to that of tubulin. Each bar represents the mean relative expression of LRF±SE of three independent experiments.

LRF has been shown to be a specific transcriptional repressor of p19ARF [9]. The transient over-expression of miR-20a shows that 48 hours post transfection the level of LRF mRNA was almost unchanged, while it was reduced following a si-RNA specific for LRF, si-LRF, (Figure 2a). p19ARF was more strongly up-regulated by si-LRF than by miR-20a at both the mRNA (Figure 2a) and protein (Figure 2b,c) levels in agreement with the degree of LRF inhibition. Downstream of p19ARF, p53 expression level was slightly increased while p21 level remained similar to control cells (Figure 2b,c).

### miR-20a over-expression decreases cell proliferation and induces senescence

**Figure 3:**
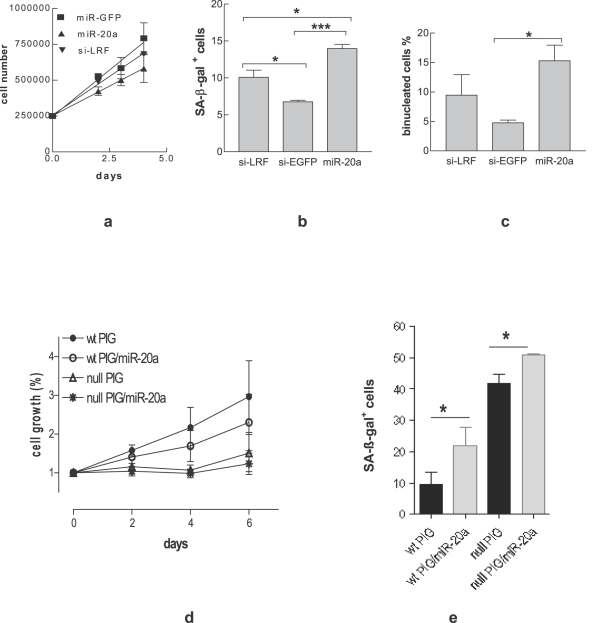
miR-20a induces senescence in MEF. Detection of cell proliferation (a), SA-ß-gal positive (b) and binucleated (c) cells in wt MEF transfected with miR-20a. Detection of proliferation (d) and SA-ß-gal positive (e) cells in wt and LRF-null MEF retrovirally infected with PIG/miR-20a. Each bar represents the mean±SE of three independent experiments.

The p19ARF up-regulation is known to inhibit MEF proliferation and to trigger senescence [9]. For this reason we investigated the effect of miR-20a on these two biological end points in MEF at early passages. MEF transfected with miR-20a proliferated less than those transfected with control miRNA (Figure 3a). To evaluate senescence, the number of SA-β-gal positive and binucleated cells was determined 96 hours post transfection. miR-20a significantly enhanced SA-β-gal positive (Figure 3b) and binucleated (Figure 3c) cells over control level. It can be noted that si-LRF was less efficient than miR-20a despite the stronger induction of p19ARF.

To reinforce the finding that miR-20a induced senescence, LRF positive and null-LRF MEF were infected with a retrovirus expressing miR-20a (PIG/miR-20a) in order to achieve a stable expression. The results show that PIG/miR-20a markedly inhibits cell proliferation (Figure 3d), and it increases the percentage of SA-β-gal positive cells two fold over control (Figure 3e). Interestingly, PIG/miR-20a is still able to cause a statistically significant increase in SA-β-gal positive cells in LRF-null cells (Figure 3e). These data and data from transient trasfection experiments, showing that miR-20a over-expression induces a greater percentage of senescent cells than si-LRF (Figure 2e), indicate that although LRF is target of miR-20a, the modulation of other targets contributes to the senescent effect.

### miR-20a over-expression affects the expression of p16INK4a and E2F1

**Figure 4:**
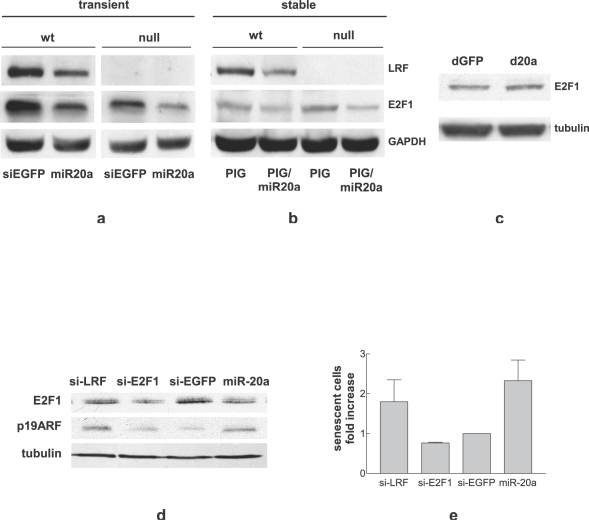
miR-20a regulates E2F1. Western blot analysis showing the expression of LRF and E2F1 in wt (a) and LRF-null MEF (b) retrovirally infected with PIG/miR-20a; c: Western blot analysis showing the expression of E2F1 in MEF transfected with d-20a; d: Western blot analysis showing the expression of E2F1 and p19ARF in wt MEF transfected with si-LRF, si-E2F1, si-EGFP and miR-20a; e: percentage of SA-ß-gal positive cells in wt MEF transfected with si-LRF, si-E2F1, si-EGFP and miR-20a.

E2F1, which plays a crucial role in senescence, is a known target of miR-20a. For this reason, the expression of E2F1 after miR-20a transient over-expression in MEF or stable expression in wild type and LRF-null MEF was determined. The results clearly indicate that miR-20a decreases E2F1 protein level in both cases (Figure 4 a,b). The depletion of the endogenous miR-20a with d-20a slightly enhanced the expression of E2F1 thus confirming that E2F1 is potentially under miR-20a control (Figure 4c). [20], [21]

We therefore asked whether E2F1 down-regulation *per se* contributed to senescence. MEF were transfected with si-E2F1: the expression of E2F1 was reduced but the reduction was not accompained by p19ARF upregulation (Figure 4d). In agreement with this result senescent cells were not induced (Figure 4e) demonstrating that E2F1 down-regulation is not enough *per se* to induce senescence.

**Figure 5:**
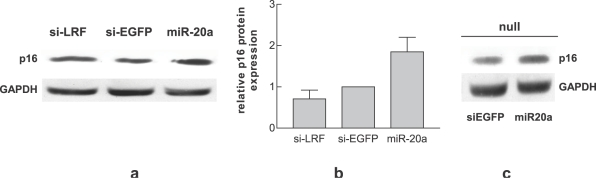
miR-20a regulates p16. Western blot analysis showing the expression of p16 after miR-20a transfection in wt MEF (a,b) and in LRF-null MEF (c). Each bar represents the mean±SE of three independent experiments.

We asked whether another key senescence inducer, the tumor suppressor p16, might be induced by miR-20a over-expression. We found that only miR-20a and not si-LRF is able to increase p16 protein levels (Figure 5 a,b). Moreover, transient miR-20a transfection in LRF-null MEF demonstrates that miR-20a is able to induce p16 also in the absence of LRF (Figure 5c).

## Discussion

The persistent mitogenic stimulation during the propagation in culture induces senescence in MEF [26]. Senescence is also induced by cellular stresses such as the over-expression of single oncogenes [27], free radicals [28], or DNA damaging drugs [29]. While the culture induced senescence occurs after 5–6 passages in culture, the stress-induced senescence appears 48–72 hours after the insult. The induction of both types of senescence is mediated by the transcriptional activation of the INK4a locus and the consequent increase of the two encoded proteins p19ARF and p16INK4a [30], [31]; p19ARF protein up-regulates p53 levels by inhibiting MDM2 [10] while p16INK4a inhibits cyclin dependent kinases thereby activating the retinoblastoma protein [32]. Moreover p19ARF has been reported to associate with proteins other than MDM2 and to have p53-independent activities most of which remain to be elucidated [30].

The transcriptional repressor LRF prevents the senescence program in MEF by inhibiting p19ARF transcription [9]. From an *in silico* analysis we found that *zbtb7a* 3′UTR is predicted to be the target of several miRNA families, among which is miR-17 family (Figure 1a). Using a hybrid reporter assay, we were able to demonstrate that miR-17 family members, in particular miR-20a, interact directly with *zbtb7a* 3′UTR (Figure 1d,e,f,g). We also over-expressed miR-20a in MEF and found that LRF protein was consistently decreased (Figure 1h), while the mRNA level was unchanged (Figure 2a). Viceversa the depletion of the endogenous miR-20a by the antisense d-20a increased LRF protein level (Figure 1i). These results clearly demonstrate that in MEF LRF is under miR-20a control.

Interestingly, we observed that the transient and stable miR-20a over-espression direct MEF toward senescence (Figure 3b,e), more similar to stress rather than to culture induced senescence, as it is accomplished within few days from transfection.

It is well known that miRNAs modulate the expression of several targets at once and in this way they activate or inhibit pathways [33], [34]. The final output depends on the fine-tuning of different players of the pathways. The miR-20a-induced senescence in MEF is not an exception to this rule. The results obtained following the stable and transient expression of miR-20a indicate the important role of LRF down-regulation/p19ARF up-regulation, but also suggest that other targets are involved. This assumption is based on the finding that miR-20a is a more powerful senescence inducer than si-LRF, although it is a milder inhibitor of LRF/inducer of p19ARF (Figure 2c) and that in LRF-null MEF additional senescence is induced (figure 3e).

Looking for other possible miR-20a targets, we focused our attention on E2F1, already known to be under miR-17 family control [20], [21], [23]. We confirmed that, when miR-20a is over-expressed either by transient transfection (Figure 4a) or stable infection (Figure 4b), E2F1 is invariably decreased. Furthermore, when endogenous miR-20a is depleted by decoy, E2F1 protein level is slightly increased confirming that E2F1 is under miR-20a control in MEF (Figure 4c).

The role of E2F1 in senescence is controversial. p19ARF is under the transcriptional control of E2F1 [35] and this explains how premature senescence can be induced by oncogenic stresses, among which E2F1 over-expression [36]. Nonetheless, in an E2F1-null setting, proper stimuli can still induce p19ARF and senescence [10]. Moreover, p19ARF can in turn decrease E2F1 protein level/activity [37], [38], [39]. Also, in some cellular context the down-regulation of E2F1 is a necessary prerequisite for senescence [40], [41]. In agreement with an anti-senescence role of E2F1, we found that si-LRF transfection slightly increases MEF senescence and induces a decrease of E2F1 (Figure 4d), possibly p19ARF-dependent. On the other hand miR-20a was able to induce a more profound E2F1 down-regulation than si-LRF both in wt and in LRF-null cells (Figure 4a,b), possibly by direct binding of this microRNA to the 3′UTR of E2F1 mRNA. These data strongly suggest that miR-20a induced premature senescence is due to the cooperation of LRF and E2F1 down-regulation. This hypothesis is supported by the finding that E2F1 knock-down by si-E2F1 *per se* is not enough to induce senescence (Figure 4e) and by the significant increase of senescence in LRF-null MEF over-expressing miR-20a concomitant with E2F1 reduction (Figure 3e). We also found that miR-20a increases another important senescence player, p16 [42], in both wild type (Figure 5a,b) and LRF-null MEF (Figure 5c). Recently the p16 pathway has been shown to have a central role in induction of premature senescence establishing an autonomous activation of ROS production via inhibition of E2F1 activity [28]. This circuit leads at the end to a cytokinesis block due to PKC activation [28] and a inhibition of mitotic exit network kinase, WARTS [43], [44]. Although we have not investigated these activities, the significant induction of binucleated cells by miR-20a (Figure 3c) and the decrease in E2F1 level are consistent with the activation of the p16 pathway as well. LRF down-regulation does not seem to play a role in p16 induction, because it has been previously reported to specifically inhibit p19ARF, but not p16 transcription [9]. We observed that, as opposed to miR-20a, si-LRF causes only p19ARF (Figure 2c) and not p16 (Figure 5a) up-regulation. Furthermore, p16 is induced by miR-20a in both wt (Figure 5a) and LRF-null MEF (Figure 5c). A plausible hypothesis is that miR-20a directly or indirectly affects a negative regulator of p16. In this respect, it is of note that E2F1 and c-Myc are linked by a positive feedback loop [45], [46], so that miR-20a-induced E2F1 down-regulation might decrease c-Myc level. Reduced c-Myc level has been shown to trigger senescence by inducing p16 via Bmi-1 down-regulation [47]. In our case the concomitant downregulation of E2F1 accompanied by p16 up-regulation are events which cooperate with LRF down-regulation to induce senescence. The combined modulation of these different proteins is likely to be at the basis of the senescence pathways elicited by miR-20a-over-expression.

In conclusion our data demonstrate that in MEF at early passages miR-20a is able to induce cellular senescence. The direct down-regulation of LRF, with the consequent induction of p19ARF, is a key mediator of the process, but the cooperation with other pathways, represented by E2F1 down-regulation and p16 up-regulation, appears to contribute. The finding that miR-20a is able to induce premature senescence is, in our opinion, interesting as it may have potential clinical relevance as anti-tumorigenic drug.

## Materials and Methods

### Reagents

Ultrahyb Oligo solution (*Ambion*); Nylon membrane Hybond-C extra, ECL detection kit (*Amersham Biosciences*); pEGFP-C1 plasmid (*Clontech*); mature miR-20a, si-EGFP, si-LRF (*Dharmacon*); antisense 2′-O-methyl-oligoribonucleotide against miR-20a (d-20a) (*LGTM, IFC, Pisa*); Gene Silencer® (*Gene Therapy Systems*); lipofectamine 2000, Trizol® Reagent DNAseI amplification grade, SuperScript II reverse transcriptase, Taq DNA polymerase, Dulbecco's Modified Eagle Medium-High Glucose (D-MEM-HG), fetal bovine serum (FBS) (*Invitrogen*); anti-LRF (*BIDMC*, *Boston*, *USA*); anti-E2F1 (sc-193) anti-p16 (sc-1661), anti-p21 (sc-397) (*Santa Cruz Biotechnology*, *Inc.*,); anti-p19ARF (ab80) (*Abcam*); anti-GAPDH (14C10) (*Cell Signaling*); anti-p53 (*Novo Castra*); T4 polynucleotide kinase (*NEB*); RNeasy mini kit for isolation of total RNA from animal cells, Polyfect, miScript System (*QIAGEN*); fraction V bovine serum albumin (BSA); LightCycler 480 Probes Master, Universal ProbeLibrary LNA Probes (*Roche*); X-Gal (5-bromo-4-chloro-3-indolylb-D-galactoside); anti-α-tubulin; polybrene (*Sigma*); pCMV-MCS plasmid, Herculase DNA polymerase, QuikChange II XL Site-Directed Mutagenesis Kit (*Stratagene*); poly-D-Lysine coated dish (*BIOCOAT, BD*); ABI PRISM 7700 Sequence Detection System (*Applied Biosystems*).

### Cells and culture conditions

Wt and LRF-null mouse embryonic fibroblasts (MEF) were isolated from 13.5d mouse embryos. Briefly, embryos were mechanically fragmented and then incubated with trypsin (0.25% in PBS pH 7.5) at 37°C for 15–20 minutes with a magnetic stirrer. After 10 minutes centrifugation at 290×g, pellets were resuspended in Dulbecco's Modified Eagle Medium+High Glucose (DMEM-HG) without fetal bovine serum (FBS) and centrifuged for 10 minutes at 290×g. After three washings, the cell suspension was distributed in culture dish containing complete DMEM-HG+10% FBS. Cells were trypsinized at confluency (p1). The propagation protocol 3T6 (6×10^5^ cells/100 mm diameter dish transferred every 3 days) was followed. HEK 293T and Phoenix E cells were grown in DMEM+10% FBS. All cells were grown at 37°C in a humidified atmosphere containing 6% CO_2_.

### Plasmids

The entire *mmu-zbtb7a* 3′UTR (Acc N° NM_010731) was obtained by PCR from genomic DNA. F (5′-GAG AAG CAC TTT AAG GAC GAG- 3′) and R (5′-GAT AGG AAG GCA AAG AGC A -3′) primers were used (T_a_ 57°C). The fragment was cloned downstream of EGFP ORF within pEGFP-C1 plasmid and a stop codon was inserted between the EGFP and the target fragment, so that the transcribed mRNA is a hybrid molecule but the translated protein is EGFP. The plasmid p-*zbtb7a* 3′UTR was constructed. The mutated version of this plasmid (p-*zbtb7a* 3′UTR_m_) was generated by utilizing the p-*zbtb7a* 3′UTR as template and modifying the miR-20a seed binding site using the QuikChange II XL Site-Directed Mutagenesis Kit. The mutagenic primers used were: site1 (position 2764 mRNA *zbtb7a*) forward 5′-TCCCCACTTTTTAAGT*T*AG*TTTTTAGATCG-3′ and reverse 5′- GCATCTAAAAAC*TA*A*CTTAAAAAGTGGGGA-3′; site2 (position 3061 mRNA Zbtb7a) forward 5′-GTGGGGATCTTGGCAT*A*TG*GTAACTGAACGG-3′ and reverse 5′-CCGTTCAG TTAC C*AT*A*TGCCAAGATCCCCAC-3′, where the asterisks indicate the mutated bases.

Genomic fragments of about 500 bp which contained human pri-miR-17, pri-miR-20a, pri-miR-106a, pri-miR-106b or pri-miR-26a sequences were obtained by PCR. Primers were: F (5′-TTTGGAACTTCTGGCTATTG-3′) and R (5′-GGCTGCAAACACAACTA-3′) for pri-miR-17; F (5′-AGTCGTCGGTCAGTCG-3′) and R (5′-CAAACCTGCAAAACTAACCATA-3′) for pri-miR-20a; F (5′-TGAGATTGCCAGTGTTATTC-3′) and R (5′-TAAGAAGTAGCCTGTGCG-3′) for pri-miR-106a; F (5′-GCAGCATATGTGGAGATG-3′) and R (5′-TCAGCAGTAGGTACGGTAA-3′) for pri-miR-106b; F (5′-CCACTGCTGACCCATTCT-3′) and R (5′-AAGACTCCTCGTTGCCAG-3′) for pri-miR-26a. Annealing temperatures were: 54.9°C for pri-miR-17, 54.2°C for pri-miR-20a, 57.6°C for pri-miR-106b, and 57°C for pri-miR-26a. F primers were elongated at the 5′ ends to include the GGATCC sequence and R primers were elongated at 5′ ends to include the CTCGAG sequence to create *BamHI* and *XhoI* restriction sites, respectively. The PCR products were cloned downstream of the CMV promoter within the pCMV-MCS plasmid. In this way, the expression plasmids nick-named p-miR-20a, p-miR-17, p-miR-106b and p-miR-26a were obtained.

pri-miR20a was amplified by PCR from genomic human DNA using the following primers: F 5′–TATTTCCTTCAAATGAATGAT-3′ and R 5′-TTCAGTAACAGGACAGTTTGA-3′. The sequence was then subcloned into MSCV-PIG [9] using BglII and XhoI (PIG/miR-20a).

### RT-PCR

To detect the expression of pre-miRNAs, total RNA was extracted from MEF using the RNeasy mini kit. After DNase treatment, 1 µg RNA was retrotranscribed using SuperScript II reverse transcriptase. The primers were those used for cloning. 2 µl of the RT mixture was then added in 50 µl PCR reactions and 7 µl aliquots were loaded on gel.

### Northern blot

20 µg of total RNA, extracted using the Trizol reagent, were loaded onto a 15% polyacrylamide 7 M urea gel, electrophoresed and successively electroblotted onto Hybond N^+^ membrane. The oligonucleotide used as probe was the complementary sequence of the mature miR-20a (miRNA Registry): (5′-CTACCTGCACTATAAGCACTTTA-3′). Probes were end-labelled with [γ-^32^P]ATP (300 Ci/mmole) by T4 polynucleotide kinase. Prehybridization and hybridization were carried out in Ultrahyb Oligo solution containing 25 µg salmon sperm DNA and 10^6^ cpm/ml labelled probes at 37°C overnight. Washing was performed in 6×SSPE at 37°C. As a loading control, membranes were stripped and re-hybridized with valine tRNA probe (5′-GAACGTGATAACCACTACACTAC-3′) or U6 probe (5′-TGTGCTGCCGAAGCAAGCAC-3′). The image of Northern hybridization signals was obtained using a Phosphoimager (B4312 Cyclone, Packard).

### EGFP reporter assay

HEK 293T cells were seeded at a density of 6×10^5^ cells per 30 mm diameter dish. 24 hours later, 140 ng of p-*zbtb7a* 3′UTR were cotransfected with 75–300 ng of p-miR-17, p-miR-20a, p-miR-106 (specific miRNAs) or p-miR-26a not predicted by any algorithm to target the 3′UTR of LRF. The aspecific miRNA was used to normalize the fluorescence values. Polyfect was used as transfectant, according to the manufacturer's recommendations. 36 hours after transfection fluorescence was quantitated by cytofluorimetry (FACSCalibur, Becton Dickinson). 10^4^ cells per sample were analysed.

### MEF transfection

Exponentially growing MEF at passage 2 were transfected with miR-20a, d-20a, siRNA-LRF, a siRNA specific for LRF (5′-CAUAAAGAAGAGUGGGAAG-3′) or si-E2F1 (Dharmacon). Briefly, 15 µl Optimem and 25 µl transfection buffer plus 80 nM miRNA or d-20a were mixed with a solution of Gene Silencer (5 µl) plus Optimem (25 µl). After 15 minutes incubation, Optimem was added up to 800 µl. Then, the transfection mixture was added to 1.2×10^6^ cells resuspended in 200 µl Optimem. After 5 min, 10 ml Optimem was added and the suspension distributed in culture dishes at a density of 2×10^4^ cells/cm^2^. After 6 hours the medium was replaced with complete DMEM-HG. With this protocol more than 90% of MEF were transfected (data not shown). Cells were collected at specified time points after transfection and further analyzed.

### MEF retroviral infection

For retrovirus-mediated gene transfer, Phoenix E cells (3×10^6^) were plated in a 100 mm poly-D-Lysine coated dish and, 16 hours later, were transfected with retroviral plasmid (PIG/miR-20a) using Lipofectamine 2000. 48 hours later, the virus-containing medium (10 ml) was filtered, mixed with 5 ml of freshly prepared medium and supplemented with 4 µg / ml polybrene. 7×10^5^ MEFs at passage 2 were plated in a 100 mm dish. 16 hours later, the medium was replaced with viral supernatant. Puromycin (2 µg /ml) was administered 48 hours after infection. The cells were subsequently selected for 2 days and then utilized for the various assays.

### Western blot

Transfected cells grown for 48 hours in 100 mm diameter dishes were collected, centrifuged and lysed (20 mM Tris-HCl pH 8.0; 20 mM NaCl; 10% glycerol; 1% NP40; 10 mM EDTA; 2 mM PMSF, 2.5 µg/ml leupeptin). Infected cells were collected immediately at the end of antibiotic selection. Proteins (30 µg/lane) were separated on 12% SDS-polyacrylamide gel and transferred to nitrocellulose membrane.

Immunoblotting of the membranes was performed using the following primary antibodies: anti-LRF (1∶1000); anti-E2F1 (1∶1000); anti-p16 (1∶250); anti-p21 (1∶1000); anti-p19ARF (1∶1000); anti-p53 (1∶1000); anti-α-tubulin (1∶20000); anti-GAPDH (1∶10000). Signals were revealed after incubation with recommended secondary antibody coupled to peroxidase by using enhanced chemiluminescence. Scanned images were quantified using Scion Image software.

### Real-time PCR analysis

To evaluate mouse *p19ARF*, *p21*, and *zbtb7a* mRNA levels, total RNA was extracted from transfected MEF using Trizol reagent according to the manufacturer's instructions. DNase treatment and retrotranscription were performed as described above. Real-time PCR was carried out using ABI PRISM 7700 Sequence Detection System (Applied Biosystems) and LightCycler 480 (Roche). Taqman probes and oligonucleotides used were as follows: for *p19ARF*, F (5′-CATGGGTCGCAGGTTCTTG-3′), R (5′-GCTCGCTGTCCTGGGTCTC-3′) and probe (5′-CACTGTGAGGATTCAGCGCGCG-3′); for *LRF*, F (5′-AACTACGACCTGAAGAACCACATG-3′), R (5′-AGATGGTCGGAGCGCACA-3′) and probe (5′-CTGCGGCCATACCAGTG CGATAGC-3′); for *p21*, F (5′-TCCACAGCGATATCCAG ACA-3′), R (5′-GGACATCACCAGGATTGGAC-3′) and LNA probe (5′-GGCCCTGG-3′). Relative quantitation of gene expression was performed with the comparative C_T_ method. GAPDH was amplified with the following primers: F (5′-GCCTTCCGTGTTCCTACCC-3′), R (5′-TGCCTGCTT CACCACCTTC-3′) and probe (5′-CCTGGAGAAACCTGCCAAGTATGATGACATC-3′) and used as internal standard. Mature miR-20a was quantified using the miScript System according to the manifacturer's instruction. Oligonucleotides 5′-TAAAGTGCTTATAGTGCAGGTAG-3′ and 5′-CGCAAGGATGACACGCAAATTC-3′ were used as forward primers respectively of miR-20a and U6 in the real time amplification mixtures. All reactions were performed in triplicate *Cellular read outs*.

### Cell proliferation

48 hours post-transfection, 1×10^5^ cells were seeded in a series of 30 mm diameter dish and grown for 96 hours. At 24 hours intervals cells were collected and counted. Growth curves after infection were generated by seeding 2.5 10^4^ cells per 12 well plates in triplicate for three independent experiments. Cells were subsequently fixed in paraformaldeyde at defined time points and subsequently stained with cristal violet. After lysis with acetic acid 10%, O.D. is read at 590 nm.

### Senescence-associated (SA) β–galactosidase activity

48 hours post-transfection, 4×10^4^ cells were seeded in 30 mm diameter dish and 48 hours later dishes were washed once with PBS and fixed for 3–5 minutes at room temperature in PBS containing 2% formaldehyde/0.2% glutaraldehyde. Cells were then washed three times in PBS, and incubated at 37°C with fresh SA-β–gal staining solution containing 1 mg/ml 5-bromo-4-chloro-3-indolyl P3-D-galactoside (X-Gal) (stock = 20 mg/ml in DMSO), 5 mM potassium ferrocyanide, 5 mM potassium ferricyanide, 2 mM MgCl_2_ in PBS pH6.0. SA-β–gal positive cells were scored after 48 hours by counting 500 cells with normal light microscopy. For infection experiments, cells were plated as described above (Cell Proliferation) and were scored 5 days after plating.

### Binucleated cells

48 hours post-transfection, 4×10^4^ cells were seeded in 30 mm dish and 48 hours later dishes were washed with PBS and fixed for 10 minutes with formaldehyde at room temperature. Cells were then stained with 0.1% crystal violet/20% methanol for 15 min. Binucleated cells were scored counting 500 cells by normal light microscopy.

### Statistical analysis

Data were analyzed using GraphPad prism (GraphPad Software, Inc., San Diego, CA). Statistical differences were determined by unpaired *t*-test, with values of P<0.05 considered statistically significant.
